# A Case Report on “Pearling”: Removal of Infected Objects During Circumcision

**DOI:** 10.7759/cureus.40700

**Published:** 2023-06-20

**Authors:** Raymond Xu, Dylan Wolff, Nicholas A Deebel, Keith Ballentine, Isaac Zucker, Gopal Badlani

**Affiliations:** 1 Urology, Atrium Health Wake Forest Baptist, Winston-Salem, USA; 2 Medical School, Florida International University, Miami, USA

**Keywords:** infection, sexual medicine, implants, circumcision, pearling

## Abstract

The insertion of foreign bodies underneath the skin of the penis is commonly referred to as “pearling.” Although rare, there are case reports that describe acute complications such as infection and damage to surrounding penile neurovascular structures; however, there is a paucity of data describing long-term complications and surgical management of such cases. A 43-year-old male presented with a penile abscess secondary to “pearling” five years after insertion. His abscess was drained and selected foreign objects were subsequently removed during a simultaneous circumcision procedure. This report describes a case in which surgical removal of penile foreign bodies was performed during a circumcision without the need for additional incisions. The case is unique in that it details a complication five years after initial insertion with microbiological data to guide adequate treatment.

## Introduction

The insertion of foreign bodies underneath the skin of the penis is commonly referred to as “pearling,” while other cultural terms exist across the world, including “fang muk” (Thailand), “penis marbles” (Western Europe), and “sputnik” (Russia) [1]. This practice has historical roots throughout southeast Asia, having been described in the ancient Indian text on eroticism and self-fulfillment, the Kama Sutra. However, it is unknown how ancient the practice may truly be [1, 2]. While most commonly described throughout Southeast Asian communities, it is also commonly performed by members of the Yakuza, the infamous organized crime syndicate in Japan [1,3]. It also appears to be practiced around the world, including being prevalent among the incarcerated, and a growing subculture among the body modification community in the Western world [1, 4-8]. The true incidence is unknown; however, it is not uncommon as different studies (albeit from non-representative samples) have found rates of approximately 1% in a random sample of young men in Thailand, and up to 5.8% of Australian prisoners [3, 7]. There is also a reported 22% prevalence amongst previously or currently incarcerated members of the Yakuza [1]. While the practice is commonly believed to increase the sexual pleasure of the individual's partner, a survey of sex workers found that many experienced severe discomfort or vaginal bleeding as a result of these implants [1]. As such, it raises questions about whether the potential benefits of the practice outweigh the potential risks. There remains a paucity of literature on the practice and the resulting complications with placement and long-term use.

The largest medical concern is that the majority of these foreign bodies are placed under sub-sterile conditions at best, which raises clear concerns for immediate complications including infection and damage to surrounding penile neurovascular structures. The fact that it is commonly performed with makeshift tools in jails and prisons, increases these concerns [1]. When complications arise, only case series and case reports offer guidance as to the treatment that can be offered. These reports range from oral antibiotics for post-insertion infections, to urgent surgical exploration for post-placement hematomas, drainage and removal for localized abscesses or infection, to excision and removal for delayed granulomatous inflammation, or surgical removal due to patient request [2, 5, 9-13]. However, complication rates are unknown and the ubiquity of the practice throughout history suggests that the overall complication rate is low. One of the largest case series published describing a physician’s encounter with patients with beads described a very low complication rate (e.g. 58 out of the 60 patients) and reported no complications, and many had beads for up to eight years [14]. Another case series describes incarcerated Australians who report penile implanting of foreign objects where the majority of the 118 subjects reported no complications, erectile difficulties, or penile pain from their presence or placement [7].

## Case presentation

Initial presentation

**Figure 1:**
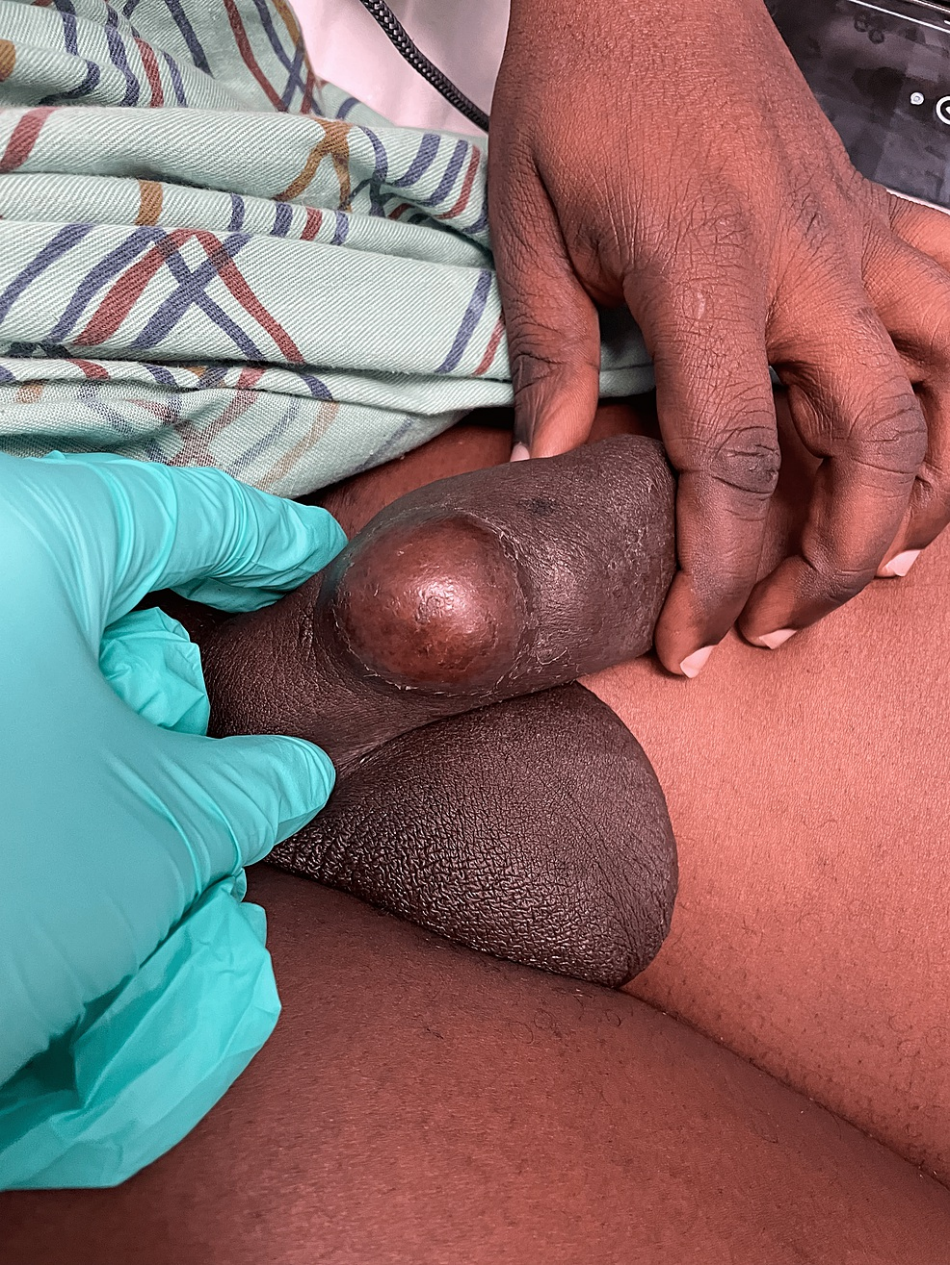
Initial emergency department presentation of penile shaft abscess

A 43-year-old, otherwise healthy, uncircumcised male presented to our emergency department with penile pain, erythema, and swelling for five days. What made his presentation notable was the presence of numerous foreign bodies under his penile skin which had been placed over several years without complications. His penile abscess was a single localized tender lesion overlying the site of several subcutaneous bodies (Figure 1). He noted his current symptoms were precipitated by intercourse 48 hours prior to the onset of his symptoms. Upon presentation, he was afebrile, hemodynamically stable, and without leukocytosis or other notable laboratory abnormalities.

**Figure 2:**
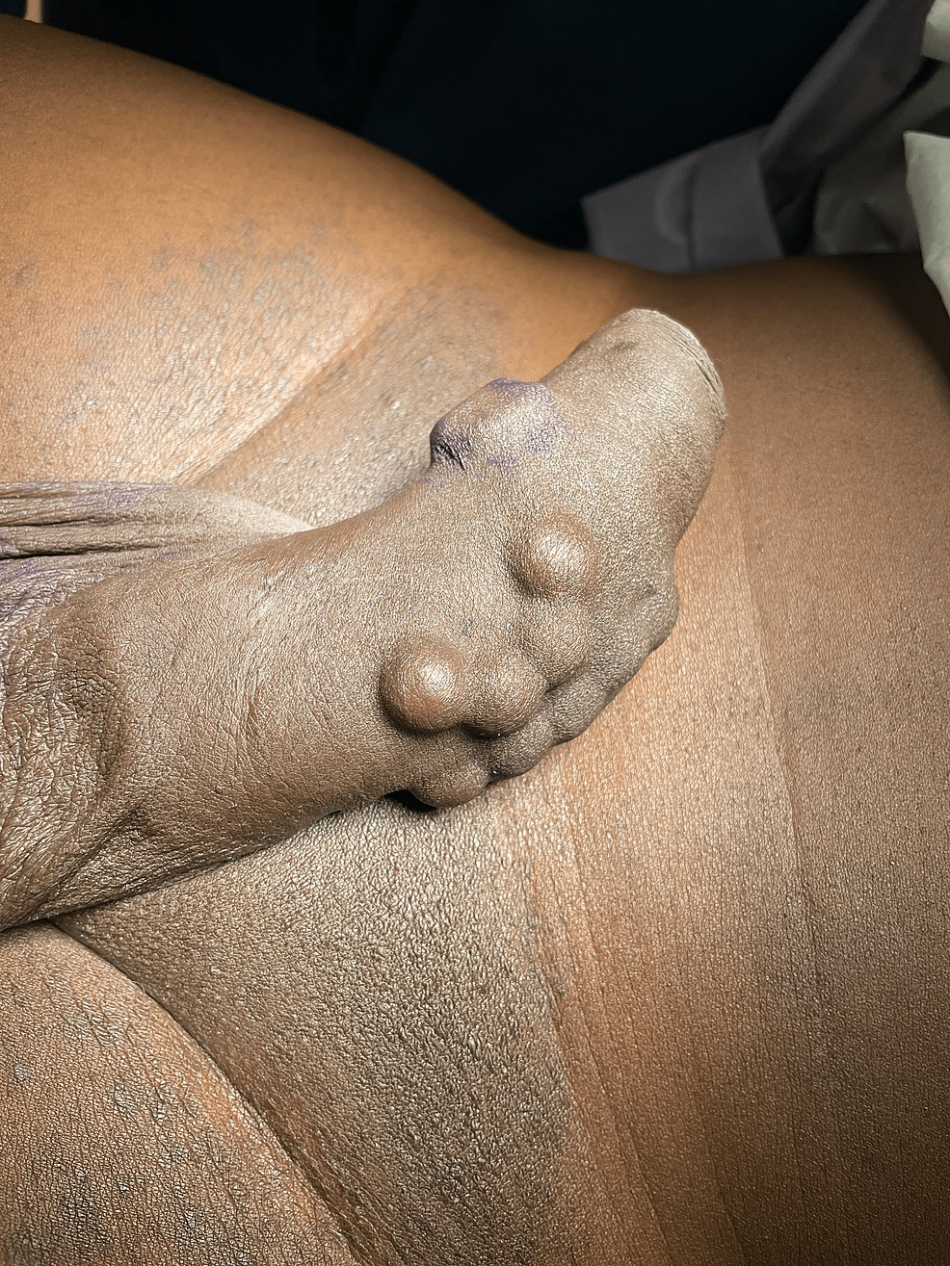
Plastic squares (left, in image) vs. metal beads (right, in image)

He first had several “beads” implanted in his home country of Haiti over seven years ago by his brother through a single incision at the base of the penis. Then, five years ago, he implanted additional plastic squares on his own through a similar process. He had not experienced any complications from these implants until he presented with the penile abscess. This was incised and drained of purulent material at the bedside, using a sterile technique in the emergency department. The purulent material was sent for culture, and he was then discharged from the emergency department on an empiric seven-day course of doxycycline. He had a follow-up appointment to discuss his preference to be circumcised, as well as our recommendation for the surgical removal of select beads/objects that had become infected. The patient requested for retention of the foreign bodies if it was medically feasible. At a two-week follow-up, his penile abscess had fully resolved. Wound cultures grew *Pseudomonas aeruginosa* sensitive to ciprofloxacin, and thus his antibiotic regimen was changed to ciprofloxacin for 14 days. The patient was scheduled for outpatient circumcision plus removal of the select “plastic squares” to reduce the risk of recurrence in the setting of an infected retained foreign body (Figure 2). The patient desired to maintain all other previously placed beads. At this time he also gave verbal informed consent for publication of the present case report.

Operative procedure

**Figure 3:**
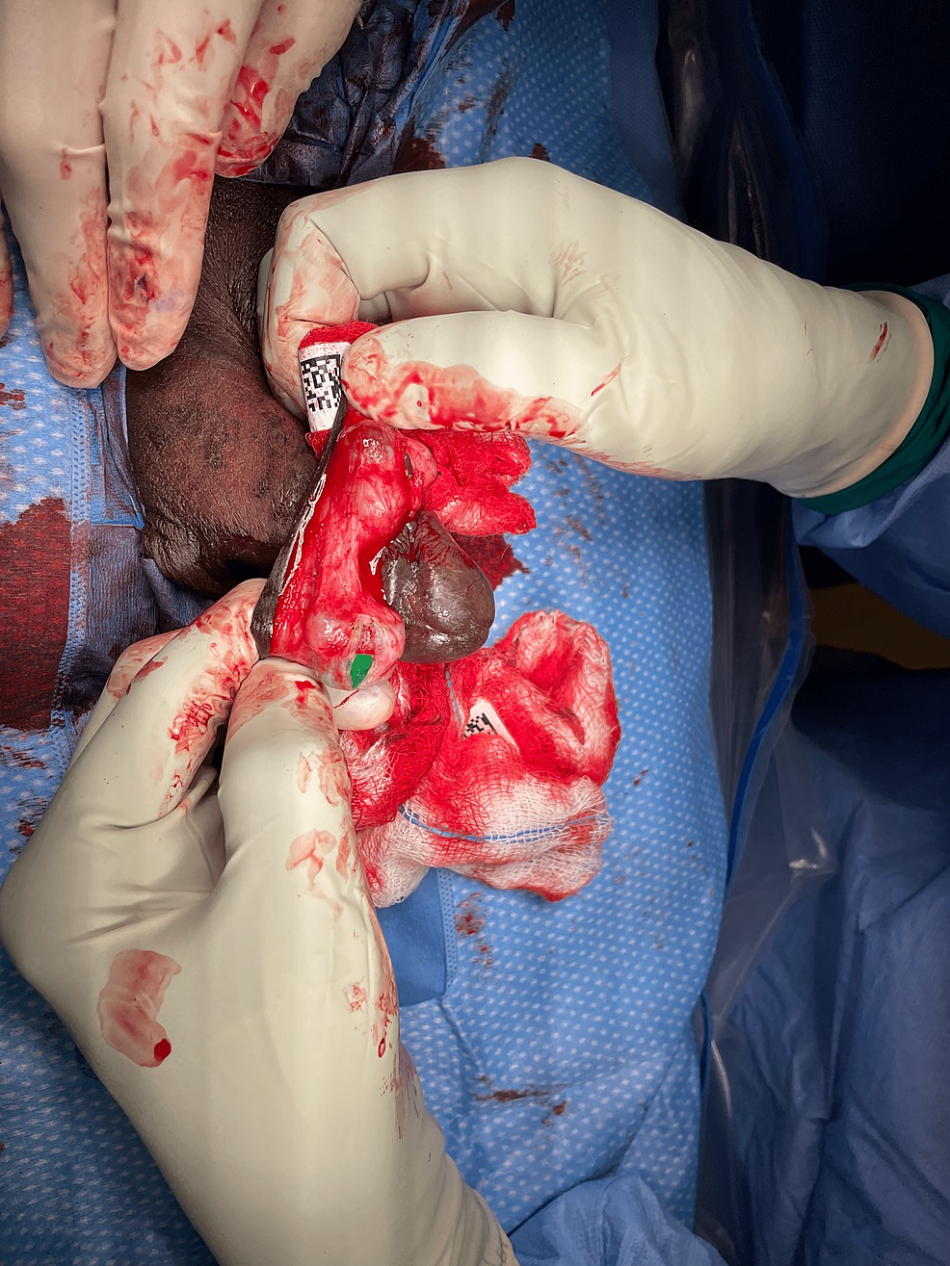
Removal of plastic squares through a proximal circumferential incision

**Figure 4:**
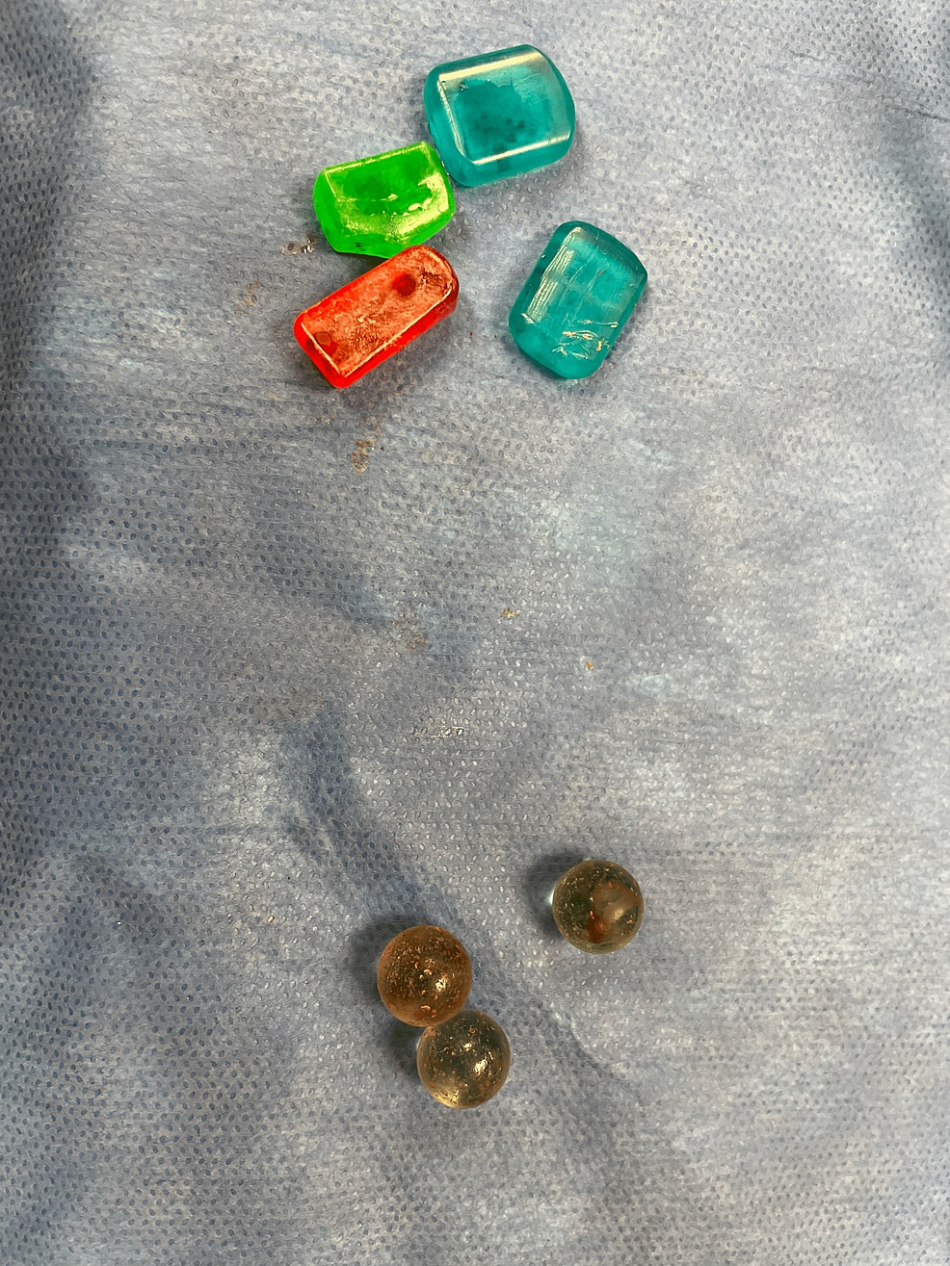
Seven total foreign objects removed: plastic squares (4) vs. beads (3)

**Figure 5:**
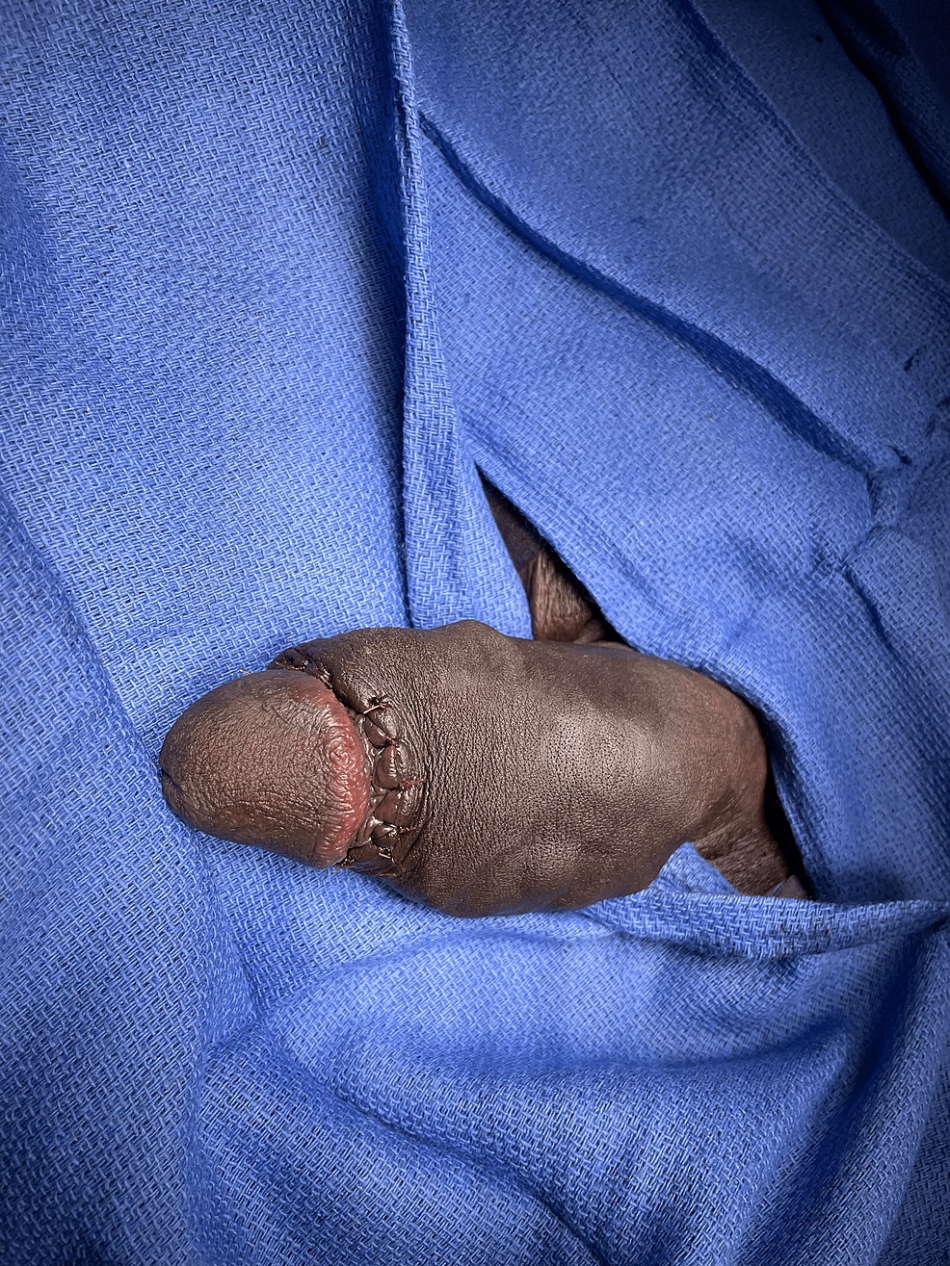
Completed circumcision with the removal of infected objects

Following normal sterile preparation and general anesthesia, the patient was draped in the operating room, the prepuce was retracted, and a circumferential incision was made on the inner prepuce approximately 1 cm proximal to the coronal sulcus. Dissection was carried down to the appropriate depth and the prepuce was returned to its native position. Similarly, a circumferential incision was made on the outer prepuce with a 15-blade scalpel, aligned with the location of the ridge of the glans. The intervening segment of skin and mucosa was then removed and Bovie electrocautery was used to obtain hemostasis. At this time, the distal four, square foreign bodies, measuring roughly 1.5 to 2.0 cm each that were selected to be removed were everted from the underside of the skin (Figure 3). They were able to be separated from Buck’s fascia and were not adherent to the tunica albuginea. An incision was made overlying the foreign bodies and they were able to be removed without difficulty. The remaining pockets from where the objects were removed were noted to have mild purulence and were irrigated with a gentamicin solution. Three glass beads were found to be too distal in the penile skin to be retained with the current circumcision incision and were thus removed (Figure 4). This pocket was also irrigated and closed with Vicryl 2-0. We then completed the circumcision by reapproximating the epithelial edges with absorbable sutures (Figure 5). A penile block was then performed with local anesthesia for postoperative pain control. The patient tolerated the procedure well and was sent home with antibiotics and a follow-up in two weeks.

Post-operative follow-up

**Figure 6:**
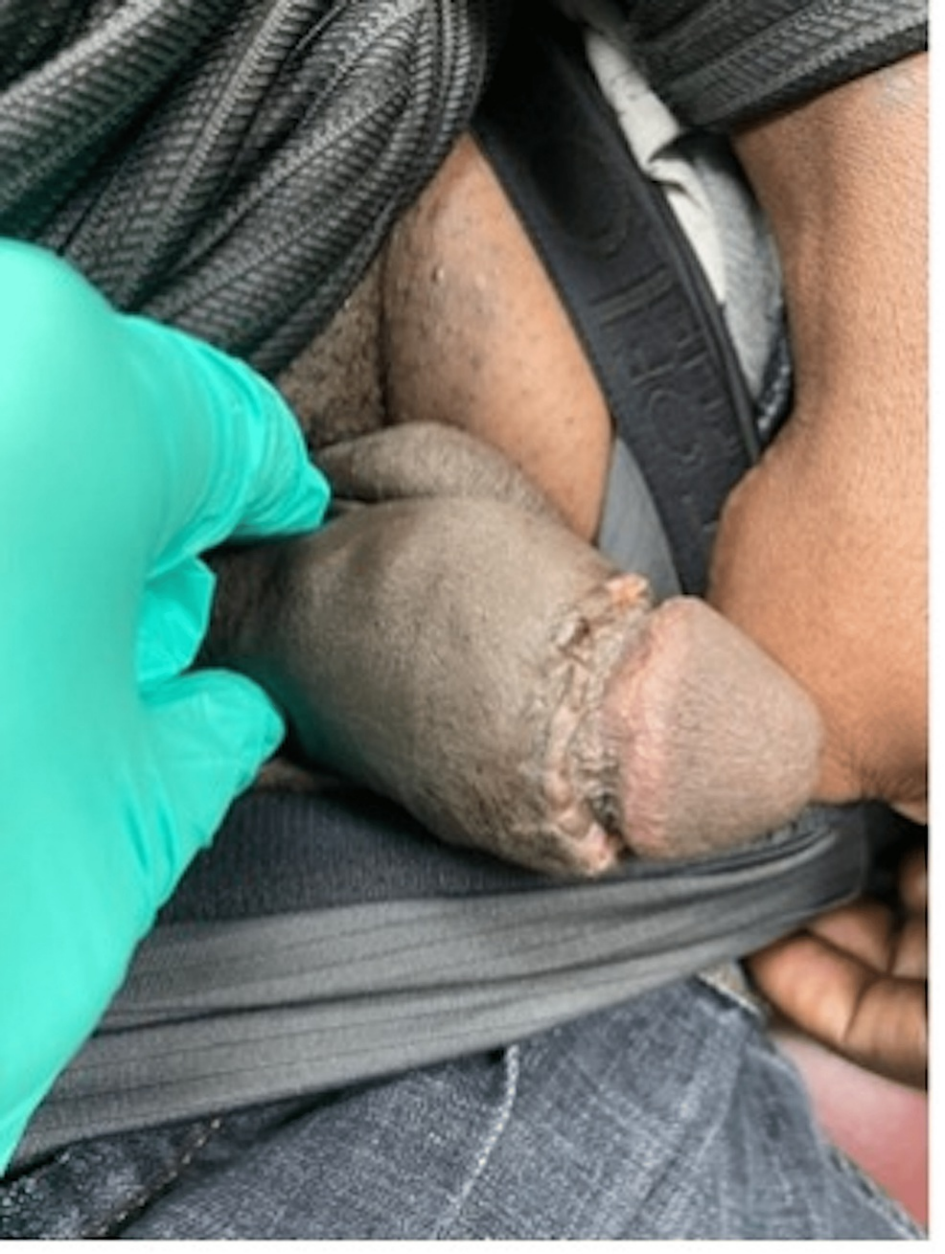
Well-healing circumcision wound two weeks post-operatively

The patient’s postoperative course was without complications. At a two-week follow-up, the circumcision incision was found to be well healing and the patient expressed great satisfaction with the cosmetic results of the procedure (Figure 6).

## Discussion

To the best of our knowledge, this represents a unique case report for numerous reasons. First, the length of time passed from the insertion of beads to the onset of infective symptoms is the longest recorded. The majority of cases reported in the literature involve immediate infection after insertion of the beads, as expected given the suboptimal sterility at the time of placement. The longest prior report in the literature for such an infection to have occurred is approximately 1 year, whereas our case represents a delayed infection five years after insertion [11,12]. We believe it is important for the medical community to be aware of the potential for long-term infective complications in these cases. The second unique aspect of our patient’s presentation involves our method of surgical removal. The patient was adamant about retaining as many of the foreign bodies as possible per the preferences of both himself and his partner, which we did our best to respect. The majority of cases involve direct removal through the skin overlying the foreign bodies through individual longitudinal or horizontal incisions [2, 5, 11]. The use of a single sub-coronal incision allowed for multiple infected foreign bodies to be removed in a manner that would not leave noticeable scars afterward, a crucial aspect of the surgery to maximize cosmetic satisfaction, and aligned with his own desire for circumcision which to our knowledge is the first description in the literature with this specific approach. This approach also provided control over which beads to remove, and which would remain in situ per the patient’s request. Three of the spherical beads were unable to be retained due to their distal location which interfered with the closure of the circumcision.

Consideration should therefore be made for a simultaneous circumcision, or degloving procedure for those previously circumcised, in surgical removal of subcutaneously implanted penile objects. While not performed in this case, this approach may also allow for cosmetically sound removal of scar tissue around the foreign objects, which could theoretically reduce the risk of unwanted sequelae such as penile curvature secondary to fibrosis.

Finally, this is the first case to include data regarding microbiology, as the initial wound culture grew *Pseudomonas aeruginosa*. The decision to use gentamicin irrigation intraoperatively was made based on the previously obtained culture sensitivities and gross purulence observed. Due to the lack of data identifying common microbial organisms in these circumstances, we suggest that a culture be obtained in all cases. 

## Conclusions

This report describes a case in which surgical removal of penile foreign bodies was performed during a circumcision without the need for additional incisions. The case is unique in that it details an infectious complication five years after initial insertion with microbiological data to guide adequate treatment.
